# Use of antibiotics in women undergoing correction of an obstetric anal sphincter injury: Results from a national Israeli survey

**DOI:** 10.1002/ijgo.14286

**Published:** 2022-06-19

**Authors:** Moshe Barg, Reut Rotem, Adi Y. Weintraub, Sorina Grisaru‐Granovsky, Rachel Michaelson‐Cohen, Misgav Rottenstreich

**Affiliations:** ^1^ Department of Obstetrics & Gynecology, Shaare Zedek Medical Center, Faculty of Medicine Hebrew University School of Medicine Jerusalem Israel; ^2^ Department of Obstetrics and Gynecology, Soroka University Medical Center, Faculty of Health Sciences Ben‐Gurion University of the Negev Beer‐Sheva Israel; ^3^ Medical Genetics Institute, Shaare Zedek Medical Center, Faculty of Medicine Hebrew University School of Medicine Jerusalem Israel; ^4^ Department of Nursing Jerusalem College of Technology Jerusalem Israel

**Keywords:** infection prevention, management protocols, obstetric anal sphincter injuries—OASIS, pelvic floor physical therapy, perineal damage, prophylactic antibiotics

## Abstract

**Objective:**

Obstetric anal sphincter injures (OASIS) have long‐term implications on women's health. Administration of antibiotic prophylaxis and treatment following OASIS repair is controversial. We conducted a national survey to provide data about practice routines regarding antibiotic prophylaxis and treatment following OASIS repair in Israeli labor and delivery units.

**Methods:**

A national survey was performed among obstetricians and gynecologists from 24 university‐affiliated delivery centers within the jurisdiction of the Israeli Ministry of Health during 2020. Representatives from each center completed the “Google form” electronic survey. For each questionnaire item, the most common answer was chosen to represent the center's answer.

**Results:**

The number of physicians who responded per center varied from 1 to 14 (median, 3.5). Preoperative and postoperative antibiotic treatment was given in 75% and 92% of the centers, respectively. While most centers (58.3%) recommend pelvic floor physical therapy on release, recommendations about functional radiologic tests vary. In all centers, there is a designated clinic for postpartum follow‐up of OASIS. Most centers (83%) allow trial of vaginal delivery in the subsequent pregnancy, on an individual basis.

**Conclusion:**

Heterogeneity exists in managing OASIS in Israel, particularly regarding administration of antibiotics. Further studies are needed to examine the consequences of different management protocols.

## Synopsis

Our national survey demonstrates that despite guidelines, management of obstetric anal sphincter injuries in Israel lacks consistency, specifically regarding administration of preoperative and postoperative antibiotics.

## 1 INTRODUCTION

Obstetric anal sphincter injuries (OASIS) refer to third and fourth‐degree perineal tears during vaginal delivery (VD) that disrupt the anal sphincter and anal mucosa, respectively.^1^ OASIS, whether overt or occult, may be the result of an otherwise uneventful VD.^2^ Rates of OASIS vary greatly worldwide, between 0.5% and 5% of VD.^3^, ^4^ It is believed that the reported rates are an underestimation^1^ of the true rates, which are much higher. OASIS may have significant and disabling future consequences on women's physical and mental health,^5^ and, as such, early recognition and proper treatment and follow‐up are of importance. In practice, guidelines regarding the recommended management during and after the repair,^6^ as well as the future follow‐up, differ among countries.^7^, ^8^ A Cochrane systematic review suggested that prophylactic administration of antibiotics is beneficial in the prevention of perineal wound infection.^9^ As a result, the American College of Obstetricians and Gynecologists (ACOG) recommends a single dose of antibiotics at the time of repair.^10^ However, an exact regimen has not been established, and there is heterogeneity in the different antibiotic regimens suggested.^11^, ^12^

The Israeli Society of Urogynecology and Pelvic Floor Medicine published guidelines on OASIS management in October 2017.^13^ These recommendations include performing the surgical repair in the operating room by a gynecologist or a general surgeon, following the administration of prophylactic antibiotics. Stool softeners are recommended in the postpartum period. In addition, the recommended follow‐up is daily during hospitalization, and at 4–6 weeks postpartum in a designated clinic. Pelvic floor physical therapy is recommended 6 weeks postpartum. Advanced radiologic tests such as transanal/transperineal ultrasound and manometry are recommended on an individual basis. Future VD may be allowed depending on the patient's preference and current symptoms and following a comprehensive discussion with the treating physician.

Despite the presence of well‐defined Israeli guidelines, it appears that not all centers in Israel follow them, and diversity in OASIS management exists. In this current national survey, we aimed to explore the variability in management and adherence with available guidelines.

## 2 MATERIALS AND METHODS

A multicenter national survey was performed among physicians from delivery units in 24 university‐affiliated medical centers across Israel. The survey was conducted during 2020. In accordance with the local institutional review board, formal ethical approval (Declaration of Helsinki) was waived, since patient information was not revealed. Representatives who treat women with OASIS from the Department of Obstetrics & Gynecology in each medical center filled out the electronic survey using a Google form (Appendix S1). In questionnaire items where an agreement between the representatives was not achieved, the most common answer was chosen to represent that medical center. The questionnaire was constructed and reviewed independently by four of the researchers (MB, RR, AYW, MR) and included items regarding prophylactic and early postpartum antibiotics, primary management and repair of the tear, and management in the early postpartum period and thereafter in subsequent deliveries. All responses were anonymized. Each question had a categorical response as well as a free‐text option.

The completed data forms were analyzed using SPSS (version 23, IBM).

## 3 RESULTS

In Israel there are 28 delivery rooms, of which 24 (86%) are university‐affiliated medical centers; these 24 centers host approximately 90% of all deliveries in the state of Israel. Physicians from all university‐affiliated medical centers with a delivery unit were surveyed and if at least one representative from each medical center responded, that medical center was included in the analysis. Non university‐affiliated delivery rooms were not approached. Overall, 200 surveys were sent and 164 were completed, setting the response rate at 82%. The number of respondents per unit varied between 1 and 14, with a median of 3.5. One center reported data for two separate units that are covered by the same physicians using the same protocols.

According to the Israeli society for Maternal‐Fetal Medicine, in 2020 the mean annual rate of OASIS tears was approximately 0.5% of vaginal deliveries (grade 3: 1.32%, grade 4: 0.21%).^14^ Specifically, the OASIS rate per 100 instrumental and spontaneous vaginal deliveries during that period were 1.9 and 0.5, accordingly.

Table 1 demonstrates the compliance with recommendations of the Israeli Society of Urogynecology and Pelvic Floor Medicine on OASIS management by center.

**TABLE 1:** Compliance with recommendations of the Israeli Society of Urogynecology and Pelvic Floor Medicine on OASIS management by center

Medical center number	No. of annual deliveries, 2020*	Rate of grade 3 to 4 perineal tears, 2020^a^	Prophylactic antibiotics before the correction of the tears	Suturing only in the operation room	A specialist performs the correction	Postpartum administration of stool softeners	Refer to pelvic floor physical therapy at discharge	Refer to follow‐up and evaluation in a designated clinic	Allow women to attempt vaginal birth after a birth with grade 3 to 4 perineal tears
1	12 500	0.38%	Yes	Yes	Yes	Yes	Yes	Yes	Yes
2	6000	0.24%	Yes	Yes	Yes	Yes	Yes	Yes	Yes
3	8300	0.50%	Yes	Yes	Yes	Yes	Yes	Yes	Yes
4	7000	0.70%	Yes	No	Yes	Yes	Yes	Yes	Yes
5	3000	1.32%	Yes	Yes	Yes	Yes	Yes	Yes	Yes
6	3900	0.25%	Yes	No	Yes	Yes	Yes	Yes	Yes
7	5800	0.30%	Yes	No	Yes	Yes	Yes	Yes	Yes
8	7000	0.60%	Yes	No	No	Yes	Yes	Yes	Yes
9	4300	0.90%	Yes	Yes	Yes	Yes	Yes	Yes	No
10	5000	0.53%	Yes	Yes	Yes	Yes	Yes	Yes	No
11	5000	0.73%	Yes	No	Yes	Yes	No	Yes	Yes
12	3800	0.10%	Yes	Yes	Yes	Yes	Yes	Yes	Yes
13	3000	0.07%	Yes	No	Yes	Yes	Yes	Yes	Yes
14	3900	0.50%	Yes	Yes	Yes	Yes	Yes	Yes	Yes
15	6500	0.68%	Yes	Yes	Yes	Yes	Yes	Yes	Yes
16	7000	0.50%	Yes	No	No	Yes	Yes	Yes	Yes
17	10 200	0.45%	No	No	No	Yes	Yes	Yes	Yes
18	2700	0.32%	Yes	No	Yes	Yes	Yes	Yes	No
19	16 500	0.35%	Yes	No	No	Yes	Yes	Yes	Yes
20	3000	0.93%	Yes	No	Yes	Yes	Yes	Yes	No
21	4700	0.55%	Yes	No	Yes	Yes	Yes	Yes	Yes
22	4000	2.10%	Yes	Yes	Yes	Yes	Yes	Yes	Yes
23	10 300	0.73%	Yes	No	No	Yes	Yes	Yes	Yes
24	15 800	0.75%	No	No	Yes	Yes	Yes	Yes	Yes
Total compliance, %			91.7	41.7	79.2	100.0	95.8	100.0	83.3

*Note*. OASIS indicates obstetric anal sphincter injures.

https://ismfm.mednet.co.il/2021/12/167165/.

^a^ Taken fron the Israeli society of fetal‐maternal medicine, Summary of 2020. Accessed December 22, 2021.

**FIGURE 1:**
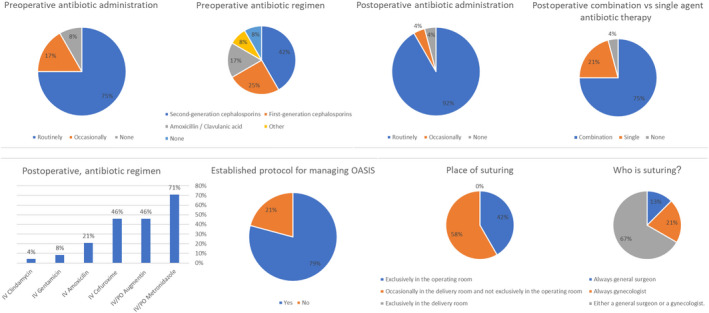
Summary of the questionnaire's results regarding antibiotic administration and primary surgical correction. IV indicates intravenous; OASIS, obstetric anal sphincter injures; and PO, orally

Our study assessed a few fundamental domains in the management and treatment of OASIS. Figure 1 summarizes the questionnaire's results regarding antibiotic administration and primary surgical correction.

### 3.1 Antibiotic administration

#### Preoperative

Before surgical repair (preoperative), antibiotics are routinely given in 18 (75%) of the centers, occasionally in four (16.7%) of the centers, and are not administered in two (8.3%) of the centers. The most prevalent antibiotic regimen was second‐generation cephalosporins (47.6%) followed by first‐generation cephalosporins (28.6%), and lastly amoxicillin/clavulanic acid (10%).

#### Postoperative

In 22 (91.7%) of the centers, antibiotics are given routinely, in one (4.2%) center they are given occasionally, and in one (4.2%) they are not given at all. In 16 (75%) of the centers administering antibiotic, a combination of antibiotics is administered. The most prevalent antibiotic is metronidazole, and the most prevalent regimen is intravenous cefuroxime in addition to oral metronidazole.

### Primary surgical correction

In 19 of 24 (79.2%) of the medical centers there is an established protocol for managing OASIS. Regarding the formal correction of the tear, in 14 (58.3%) of the centers, the surgical correction of the tear is performed occasionally in the delivery room and not exclusively in the operating room. In addition, in three (12.5%) of the centers, the correction is always performed by a general surgeon and in five (20.8%) of the centers it is performed always by a gynecologist. In the remaining 16 (66.7%) centers, the correction may be performed by either a general surgeon or a gynecologist.

**FIGURE 2:**
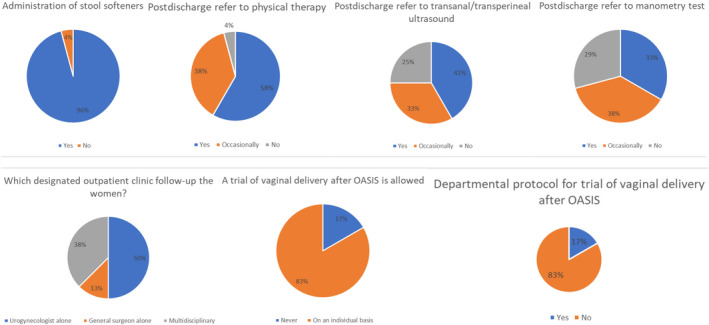
Summary of the questionnaire's results regarding the early and late postpartum management of women with 3–4 perineal tear. OASIS indicates obstetric anal sphincter injures

Figure 2 summarizes the questionnaire's results regarding the early and late postpartum management of women with OASIS.

### Stool softeners

In almost all of the centers (23 [95.8%]), stool softeners are recommended during the postpartum period.

### 3.2 Physical therapy and anatomic and functional imaging

When discharged from the hospital, 14 (58.3%) of the centers routinely refer women to pelvic floor physical therapy, nine (37.5%) of the centers do so occasionally, and one (4.2%) center does not provide recommendations. In addition, 10 (41.7%) of the centers routinely recommend a transanal/transperineal ultrasound during the postpartum visit at 6 weeks and beyond, while eight (33.3%) give this recommendation occasionally and six (25%) do not recommend this at all. Finally, eight (33.3%) of the centers routinely recommend performing an anal manometry test during the postpartum visit at 6 weeks, while nine (37.5%) of the centers do so occasionally and seven (29.2%) do not recommend performing an anal manometry test.

### 3.3 Postpartum visit

In virtually all 24 centers, a designated outpatient clinic follows patients with OASIS during the postpartum period. In 12 (50%) of the centers, the follow‐up is conducted by a urogynecologist alone and in three (12.5%) of the centers, the follow‐up is conducted by a surgeon alone. In the remaining nine (37.5%) centers, the clinic is multidisciplinary and includes an urogynecologist, a surgeon, and occasionally a gastroenterologist.

### 3.4 Subsequent deliveries

In four (16.7%) of the centers, a trial of VD is never recommended following an OASIS, while in the remaining 20 (83.3%) centers it may be allowed on an individual basis. When allowing a vaginal delivery, four (16.7%) of the centers make the decision based on a departmental protocol, while 20 (83.3%) of the centers do not have a strict protocol.

## 4 DISCUSSION

This national survey among physicians from 24 delivery units across Israel regarding the management of OASIS has demonstrated great diversity in the antibiotic regimens and the routine postpartum practice in Israel. This is despite clear national guidelines that were published 3 years before this survey.

A few surveys regarding management of OASIS have been previously published. In 2002, Fernando et al. reported the results of questionnaires that were sent to 672 coloproctologists, consultant obstetricians, and obstetric trainees in the UK. Perioperative antibiotics were recommended by 91%, 79%, and 88%, respectively. Stool laxatives were advised by 78%, 94%, and 82%, respectively. Coloproctologists suggested a follow‐up period of >12 months, compared with most obstetricians, who suggested 6 weeks only. With regard to subsequent delivery, most coloproctologists recommended cesarean delivery, while most obstetricians allowed a vaginal delivery. The majority of OASIS repairs were performed in the delivery room (89.4%) as opposed to the operating room.^15^ In an additional survey that was taken in the UK in 2009, OASIS repairs were performed in the delivery room in most cases (78.8%). Prophylactic antibiotics were prescribed to 98.1%, laxatives to 95.2%, and pelvic floor muscle training was recommended in 71.2% of cases.^16^ In a survey of clinical practice from Canada, 51.1% of physicians routinely ordered intraoperative prophylactic antibiotics during repair, 97.2% of the survey respondents routinely ordered laxatives or stool softeners, and 28.9% ordered a course of postoperative antibiotics. Eighty‐four percent of respondents indicated that they routinely arranged follow‐up for women after OASIS.^17^

In Israel, surgical correction may be conducted in the delivery or operating room, with most units correcting the tear in the delivery room. In published guidelines by the American College of Obstetrics and Gynecology (ACOG),^8^ it is not specified where surgical correction should take place, while in guidelines by the Royal College of Obstetricians & Gynecologists (RCOG)^7^ it is specially stated that the correction should be performed in the operating room.

Regarding antibiotic administration, in most Israeli centers, preoperative and postoperative antibiotics are given (75% and 92%, respectively). The ACOG encourages the administration of preoperative antibiotics and states that postoperative administration should be further studied.^8^ RCOG^7^ recommendations include administration of postoperative antibiotics.

Stool softeners are recommended by both the ACOG and RCOG.^7^, ^8^ On release from the hospital, most units in Israel recommend pelvic floor physical therapy, while the recommendation for advanced functional imaging tests is not widely recommended. The RCOG states that performing pelvic floor physical therapy may be beneficial,^7^ while the ACOG states that the durable long‐term effect of this intervention has yet to be studied and does not recommend it on a routine basis.^8^ Postoperative follow‐up is not specified in the guidelines provided by the ACOG,^8^ and the RCOG recommend follow‐up at 6–12 weeks postpartum, preferably by clinicians with substantial experience in OASIS care. Lastly, in the majority of delivery units, a trial of VD is considered on an individual basis. Both the ACOG and RCOG state that the management of future deliveries depends on both symptomatic and clinical evaluation, and the decision should be individualized to the patient.^7^, ^8^

The adherence to guidelines and protocols among different fields of medicine has been previously studied and many systems have been created to reinforce medical adherence to the current published literature and to provide patient‐centered and consistent care.^18^, ^19^, ^20^ The main motivation for standardized care stems from the understanding that different treatment regimens may have different effects on a patient's well‐being. In this study, similar to studies in other fields, we have demonstrated that OASIS care in Israel lacks the consistency that is expected when providing medical care in a developed country. We assume that this reflects the situation in additional developed countries. As the Israeli national health plan covers delivery care for all women, adherence to protocols is most probably not biased by financial burdens, which should facilitate standardizing care.

The implications of differences in management of OASIS have been scarcely studied.

As previously stated, the general use of antibiotics in the management of OASIS is not evidence‐based.^21^ In a single‐center study in Michigan, the implementation of a quality improvement intervention with a single dose of prophylactic antibiotics administration during OASIS repair resulted in a clinically meaningful decrease in wound infections.^22^ Of note, this study was underpowered to detect a significant difference in other wound complications. In addition, the exact regimen has not been established.^11^, ^12^ In a recently published systematic review, the use of metronidazole during OASIS repair was studied. It was found that despite its wide use during OASIS repair and recovery, this practice is not evidence‐based and was not prospectively studied.^21^ A systematic review that aimed at evaluating the evidence of routine pelvic floor physical therapy in the management of OASIS concluded that data are scarce and the level of evidence to support this intervention is currently of low quality.^23^ With regard to future modes of delivery, a multicenter randomized study from Paris, France, demonstrated that elective cesarean delivery has no advantage in preventing anal incontinence in asymptomatic women.^24^ In contrast, a different study found that even among slightly symptomatic women, VD carries a risk of deterioration of anal incontinence symptoms that is higher than in patients undergoing an elective cesarean delivery.^25^

Our survey once again highlights the lack of evidence‐based medicine in the practical and optimal management of OASIS, one of the most debilitating outcomes of childbirth. Hence, professional organizations, which aim to provide recommendations for optimal health care, need to study the outcomes of different protocols and treatment regimens. An effort should be made to create international evidence‐based guidelines on managing OASIS.

Our study has several strengths. First, as far as we know, this was the first survey to investigate this important issue. Second, this was a national study that was sent to all university‐affiliated centers in Israel and responses were collected from all of them; the departmental protocols, as well as the obstetrical practice and decision‐making processes, were examined throughout a specific study period. In addition, there were several participants from each medical center, which reduces potential recall bias.

Nonetheless, our study was not without limitations. The questionnaire was sent using social media to physicians from all university‐affiliated obstetrics and gynecology departments who care for women with OASIS. If at least one physician responded from a medical center, that medical center was included in the analysis. The study was sent to many physicians who care for women with OASIS and not all of them responded. As a result, the exact number of the physicians who received the questionnaire remains unknown. Since the study was anonymous, we were not able to characterize those who did not answer, and hence a potential selection bias may exist. However, our aim was to assess the practice routines in Israeli labor and delivery units and not among different physicians. Moreover, as the response rate was high and answers were diverse, we believe that this would be a nondifferential bias and hence negligible. Additionally, in this study we did not address the actual mode of surgical repair (eg, end‐to‐end or overlap, sutures type) and hence are not able to provide data on this matter. In addition, in some questions, the answers varied within a single medical center, highlighting not only the inter‐unit but even more so the potential intra‐unit variation. As stated above, the most prevalent answers were selected; there were no cases of similar rates of two different answers in none of the centers. Lastly, many of the responders in the study were not those who repair the tears; nonetheless, all of the responders were involved in the management of the cases in the delivery rooms, during the early postpartum period, and in the discharge of these women, providing them with specific recommendations; therefore, they could reliably provide answers to the questions asked.

In conclusion, our national survey demonstrated that despite clear national guidelines, the practice in Israel in the management of OASIS is diverse, specifically regarding the administration of preoperative and postoperative antibiotic treatment. This may have implications on women's future health, well‐being, and satisfaction. More studies are needed to compare outcomes resulting from different regimens of antibiotics and to create standardized guidelines accordingly.

## AUTHOR CONTRIBUTIONS

MB designed, planned, and conducted the study, and wrote the article. RR designed, planned, and conducted the study, and wrote the article. AYB conducted the study. SGG assisted in planning the study. RMC analyzed the data and edited the article. MR designed, planned, and conducted the study.

## CONFLICT OF INTEREST

None of the authors declare any conflict of interest.

## Supporting information


Appendix S1

## Data Availability

Research data are not shared.
