# Supplementary material for: Use of antibiotics in women undergoing correction of an obstetric anal sphincter injury: Results from a national Israeli survey
Source: Int J Gynaecol Obstet. 2022 Jun 19;160(1):195–201. doi: 10.1002/ijgo.14286 (PMC10084148; doi:10.1002/ijgo.14286)
Supplement: Supplementary file 1 — Appendix S1 [file IJGO-160-195-s001.docx]

Appendix 1: Questionnaire on: Post-Partum treatment of grade 3/4 perineal tears

According to the guidelines of various associations, antibiotics are recommended after tears of grade 3/4. However, the timing of such treatment, its regimen, and the follow up protocols are not unified. The goal of this survey is to evaluate the different management approaches in these cases.

Hospital:

- Rambam
- Carmel
- Ha’Emek
- Bnei Zion
- Poriya
- Galil-Nahariya Medical Center
- Ziv
- Laniado
- Meir
- Hadassah Ein Kerem
- Hadassah Mount Scopus
- Shaare Zedek
- Wolffson
- Assaf Harofe
- Ma’ayaney Hayeshu’a
- Ichilov
- Kaplan
- Belinson
- Soroka
- Hilel Yafe
- Assuda Ashdod
- Barzilay
- Nazareth
- Other

Do you know of a settled protocol in your department for the post-partum treatment of grade 3/4 perineal tears?

- Yes
- No
- I don’t know

Do corrections of grade 3/4 perineal tears sometimes take place in the delivery room (and not just the operation room)?

- Yes
- No

Who performs the correction of the tear?

- Always a gynecologist
- Always a surgeon
- Sometimes a gynecologist and sometimes a surgeon

Who performs the correction?

- A specialist
- A resident
- Sometimes a specialist and sometimes a resident

 Does your department preventively administer antibiotics before the correction of grade 3/4 perineal tears?

- Yes
- No
- Sometimes yes and sometimes no

If preventive antibiotics is used before the correction of the tear, what kind is used?

- First generation cephalosporin
- Second generation cephalosporin
- Augmentin
- Other antibiotics
- It is not used

Does your department administer stool softeners post-partum with grade 3/4 perineal tears?

- Yes
- No
- Sometimes

Which antibiotics are used in post-partum treatment? If there is a departmental protocol, please write what would be used and if not, please write what you would use. You can choose multiple answers

- IV Flagyl
- PO Flagyl
- IV Ampicillin
- PO Ampicillin
- IV Cefuroxime
- PO Cefuroxime
- IV Augmentin
- PO Augmentin
- Other:

Are you familiar with a protocol in your department for post discharge follow up with women with grade 3/4 perineal tears?

- Yes
- No
- I don’t know

Are these women referred to pelvic floor physical therapy at discharge?

- Yes
- No
- Sometimes yes and sometimes no

Are these women referred to transrectal/transperineal ultrasound at discharge?

- Yes
- No
- Sometimes yes and sometimes no

Are these women referred to manometry at discharge?

- Yes
- No
- Sometimes yes and sometimes no

Are the women referred to continue their follow up and evaluation in a designated clinic? If several clinics are involved, please mark all of them

- Perineum clinic
- Urogynecology clinic
- Gastroenterology clinic
- Surgical clinic
- Interdisciplinary clinic
- They are not referred to follow up at a clinic

Does your department allow women to attempt vaginal birth after a birth with grade 3/4 perineal tears?

- Yes
- No
- Sometimes

Do you know of a protocol (with defined conditions) in your department for vaginal birth following grade 3/4 perineal tears?

- Yes
- No
- I don’t know

If such a protocol exists, which conditions does it include? Please mark all those that apply

- Weight estimate of under 3500g
- Avoiding instrumental delivery
- Shortening of second stage
- Performing an episiotomy
- Lack of symptoms related to grade 3/4 perineal tears
- Receiving confirmation before delivery from a senior physician
- Having undergone an evaluation that includes transrectal/transperineal ultrasound
- Having undergone an evaluation which includes manometry
- A mild tear – only grade 3A
